# Ferroptosis in Different Pathological Contexts Seen through the Eyes of Mitochondria

**DOI:** 10.1155/2021/5537330

**Published:** 2021-06-07

**Authors:** V. Otasevic, M. Vucetic, I. Grigorov, V. Martinovic, A. Stancic

**Affiliations:** ^1^Department of Molecular Biology, Institute for Biological Research “Siniša Stanković, ” National Institute of Republic of Serbia, University of Belgrade, Serbia; ^2^Medical Biology Department, Centre Scientifique de Monaco (CSM), Monaco

## Abstract

Ferroptosis is a recently described form of regulated cell death characterized by intracellular iron accumulation and severe lipid peroxidation due to an impaired cysteine-glutathione-glutathione peroxidase 4 antioxidant defence axis. One of the hallmarks of ferroptosis is a specific morphological phenotype characterized by extensive ultrastructural changes of mitochondria. Increasing evidence suggests that mitochondria play a significant role in the induction and execution of ferroptosis. The present review summarizes existing knowledge about the mitochondrial impact on ferroptosis in different pathological states, primarily cancer, cardiovascular diseases, and neurodegenerative diseases. Additionally, we highlight pathologies in which the ferroptosis/mitochondria relation remains to be investigated, where the process of ferroptosis has been confirmed (such as liver- and kidney-related pathologies) and those in which ferroptosis has not been studied yet, such as diabetes. We will bring attention to avenues that could be followed in future research, based on the use of mitochondria-targeted approaches as anti- and proferroptotic strategies and directed to the improvement of existing and the development of novel therapeutic strategies.

## 1. Introduction

Despite the progress in medicine during recent decades, diseases such as cancer, neurodegenerative and cardiovascular diseases, and ischemic heart and brain injury still have considerable mortality rates worldwide [1]. In particular, worrying is the fact that the incidence of these illnesses is globally on the rise, pointing to an urgent need for the development of novel and effective therapies. Although diverse treatment strategies have been exploited for each condition, a major concern that still exists is deregulated or adverse cell death in the affected tissues, the hallmark that connects these pathologies at the molecular level. Cell destiny (cell death and cell survival) is under the tight control of mitochondria [2–4]. These organelles regulate a variety of vital cellular activities, including ATP synthesis, intracellular calcium homeostasis, hormone production and responses to hormones, cell differentiation, and cell death [3]. Thus, it is not surprising that defects in mitochondrial activity and/or specific alterations in mitochondria-mediated signalling pathways are also the hallmarks of the above-mentioned diseases [5, 6]. Therefore, targeting mitochondria-mediated pathways aimed at manipulating cell death in diseases has a promising therapeutic potential and is of immense research interest. Apoptosis, as the major pathway of normal cell turnover, has been widely and extensively studied from the aspect of targeting mitochondria as a therapeutic strategy and has proven to be an effective target in many diseases (especially in cancer and neurodegenerative and cardiovascular diseases) [7–10]. Depending on the context, mitochondria-dependent apoptotic approaches can be aimed at apoptosis induction, as in cancer, or its inhibition, as in neurodegenerative and cardiovascular diseases.

Although apoptosis modulation through different strategies has achieved significant success in the treatment of these diseases [8–11], they are still far from being negligible, which suggests that new avenues for the development of clinical regimens need to be examined. One such avenue has been opened with the discovery of a new type of regulated cell death—ferroptosis.

Ferroptosis is a newly detected form of regulated cell death characterized by iron-dependent accumulation of lipid peroxides arising from the iron-catalysed oxidation of polyunsaturated fatty acids that can be pharmacologically inhibited by iron chelators and lipid peroxidation inhibitors [12]. It differs from apoptosis in the changes of cell morphology and biochemistry [12–14]. On the other side, the molecular pathways and key players involved in ferroptosis are very similar, if not identical to those involved in the induction/mediation of oxytosis or oxidative stress-induced programmed cell death as defined by Tan et al. [15] almost one decade before ferroptosis. Lewerenz et al. [16] and Maher et al. [17] have suggested that oxytosis and ferroptosis are two names for the same cell death pathway reviewing the strong evidence for their assertion. Extensive research into the process of ferroptosis during the last few years has revealed that it is linked to different pathological states, such as cancer, neurodegenerative, cardiovascular, and hematologic diseases, and ischemia/reperfusion (I/R) injury of various organs, including the heart, brain, and kidney [18–23]. Thus, modulating ferroptosis, either by targeted induction (e.g., cancer) or by its prevention (e.g., in neurodegenerative and cardiovascular diseases), might be a promising therapeutic approach, and together with apoptosis modifiers, this has contributed to better understanding of all the above-mentioned diseases and improvement of their treatment. The antipathological rationale is supported by the effects of inducers (erastin, Ras-selective lethal small molecule (RSL)) and inhibitors (ferrostatin-1, liproxstatin-1) of ferroptosis that have been developed thus far, which can successfully modulate cell death in cancer and neurodegenerative and cardiovascular diseases [22, 24]. At the same time, the ability to specifically target mitochondrial pathways to modulate ferroptosis is less exploited. The reason for this lies in the fact that in contrast to the well-defined roles of mitochondria in the regulation of apoptosis, their role in the ferroptotic type of cell death is less well defined [25, 26]. In addition, the results of studies investigating the relationship between mitochondrial functionality and ferroptosis are controversial [12, 20, 27–29], and the lack of consensus as to their actions keeps the debate open.

However, the recently revealed molecular mechanisms of ferroptosis show that the involvement of mitochondria in the regulation of this type of cell death has become more important and points to further directions in research.

Specifically, the crucial role of mitochondria in lipid peroxidation-driven ferroptotic cell death has been observed in the following conditions: (i) impaired mitochondrial metabolism and ensuing extensive production of reactive oxygen species (ROS), (ii) excess free-iron accumulation in mitochondria, and (iii) mitochondrial cysteine deprivation [27, 28, 30]. Along these lines, the prevention of these mitochondrial states significantly correlates with increased resistance to ferroptotic cell death [28, 30–32]. Great progress has been made in the protection of cardiomyocytes and neuronal cells from the inducers of lipid peroxidation and ferroptosis by blocking mitochondria-dependent ferroptosis pathways using specific mitochondria-targeted ROS scavengers, antioxidants, and modulators of mitochondrial iron levels [12, 28, 32–35]. In the reverse approach aimed at sensitizing cancer cells (e.g., hepatocarcinoma) to their demise, the anticancer activity of erastin, an inducer of ferroptosis, was mediated by mitochondrial pathways that involve voltage-dependent anion channel (VDAC) opening, mitochondrial hyperpolarization, consequent increase in ROS production, and mitochondrial dysfunction [36, 37]. In addition, since mitochondrial morphology and functional activity coexist in an intimate structure/function relationship [38–40], along with the characteristic phenotype of mitochondria in ferroptotic cells, mitochondrial function in ferroptosis is compromised [27, 31] and rescuing or damaging mitochondrial function can affect the outcome of ferroptosis.

Taking all these considerations into account in addition to the well-known contributing role of mitochondria to many different pathologies, we have reviewed the existing knowledge of the role of these organelles in the process of ferroptosis in disease states, primarily in cancer and neurodegenerative and cardiovascular diseases. We will also discuss the potential implementation of specific mitochondria-targeted approaches for modulating ferroptotic cell death beyond these diseases and in other pathological conditions that are accompanied by increased cell death and/or impaired mitochondrial function. Finally, we will present future directions in the examination of various mitochondria-targeted approaches for modulating (through either induction or suppression) ferroptosis with the aim of improving the understanding and targeted therapy of the discussed diseases.

## 2. Ferroptosis: The Basic Signalling Pathways and Its Hallmarks

Ferroptosis is an iron-dependent, lipid peroxide-driven form of cell death that differs from other forms of regulated cell death with respect to the biochemical pathways, the main triggers and executors, and morphological features. Since 2012 when the Stockwell Group first recognized ferroptosis as a new form of cell death, evidence in support of its uniqueness has increased [12]. The main event in ferroptosis is the uncontrolled accumulation of lipid peroxides as a result of an imbalance in their production and removal. Two major pathways lead to lipid peroxide formation: enzymatic and nonenzymatic [41]. The former involves enzymes that catalyse the metabolism of arachidonic acids and polyunsaturated fatty acids (PUFAs), with the central role assumed by lipoxygenases (LOXs). The latter is a redox-based mechanism of lipid peroxide production. This process is triggered when ROS, reactive nitrogen species (RNS), or reactive lipid species (RLS) extract a hydrogen atom from a PUFA, forming a lipid radical (L^∙^) [41]. The hydroxyl radical (^∙^OH) and the hydroperoxyl radical (^∙^OOH) are the strongest initiators of this process. They are formed in the reaction of ferrous iron (Fe^2+^) with hydrogen peroxide (H_2_O_2_) in the Fenton reaction [41]. Regardless of the pathways that lead to the initiation of lipid peroxidation, this process moves on to the second and third phases, propagation and termination, respectively [42]. In the propagation phase, L^∙^ rapidly reacts with oxygen to form a lipid peroxy radical (LOO^∙^), which extracts a hydrogen atom from another lipid molecule, generating a new L^∙^ (which continues the chain reaction) and a lipid hydroperoxide (LOOH) [41]. Once lipid peroxidation is initiated, propagation of chain reactions occurs until the termination products are produced. The lipid peroxides produced during the propagation phase can be converted into hydroxy fatty acids or reactive aldehydes that initiate lipid peroxidation themselves and thus the chain reaction [42].

However, it is unclear how lipid peroxidation leads to ferroptotic cell death. It is likely that cell death is the result of multiple events, including direct damage to specific membranes and the activation of downstream pathways [43–45]. Membrane lipid peroxidation induces its disruption and changes physical properties, decreasing membrane fluidity, increasing membrane permeability, disturbing the ion gradient, and slowing down lateral diffusion [46–49]. Furthermore, the formation of secondary products of lipid peroxidation of PUFAs can modulate protein functions and several signalling pathways that interfere with cell death [50, 51]. Among these, malondialdehyde (MDA) and 4-hydroxynonenal (4-HNE) are the most abundant and exhaustively studied in the context of other types of cell death, apoptosis and necrosis [52]. It was shown that these aldehydes accumulate in different models of ferroptosis [14, 53, 54], but their significance in this type of cell death needs to be defined.

Under basal conditions, the lipid peroxidation cascade is controlled by the antioxidant enzyme glutathione peroxidase 4 (GPX4) that uses glutathione (GSH) to reduce lipid peroxides to their alcohol form (LOOH). GSH-GPX4, or more precisely the axis involving cysteine-GSH-GPX4-lipid peroxides, constitutes the backbone of ferroptosis signalling [55]. GSH is a simple tripeptide with cysteine playing an important role in redox state regulation due to its reactive thiol. In addition to its role as a cofactor for GPX, two more aspects of GSH action are relevant to ferroptosis: (i) regulation of the activity and intracellular translocation of LOXs [15, 56] and (ii) the redox cycling activity of Fe^2+^ [45, 57]. Depletion of GSH increases both LOX activity and membrane translocation as well as the substrate of the Fenton reaction. Also, GSH is a cofactor for glutathione S-transferase (GST) in the reaction of detoxification of both ROS and xenobiotics [58].

Cells obtain cysteine from the extracellular environment in the oxidized (dominant) form, cystine, through the cystine/glutamate antiporter, xCT. In the cell, cystine is reduced to cysteine via cysteine reductase and “incorporated” into GSH through enzymatic reactions catalysed by glutamate-cysteine ligase (GCL) and glutathione synthetase (GSS). Consequently, one approach for ferroptosis induction involves the targeting of xCT. Erastin has been widely used as a pharmacological modulator/inducer of ferroptosis [59]. Initially, erastin was described as an antitumor compound that induces cell death in RAS-overexpressing cells [60]. Subsequently, it was reported that erastin exhibits an inhibitory activity over VDAC [37] and xCT [12]. Although both these actions of erastin are related to ferroptosis induction, it is likely that inhibition of VDAC2 and VDAC3 is necessary but not sufficient for erastin-induced death [37]. It is believed that targeting of the system xCT plays a major role in the process of ferroptosis induction by erastin [12]. However, the effect of erastin on VDAC is relevant in the context of the role of mitochondria in ferroptosis, as discussed above. Besides these pharmacological approaches that target xCT and cystine/glutamate metabolism, natural triggers that induce ferroptosis in physiological contexts have been described. One is the accumulation of extracellular glutamate [12, 20]. Glutamate-induced toxicity has been extensively studied in HT22 cells, a specifically designed mouse hippocampal nerve cell line that is particularly sensitive to glutamate and is killed exclusively via the oxidative pathway called oxytosis [61]. The first step in this cell death is glutamate-induced inhibition of cystine uptake through xCT [62]. The same pathway of glutamate-induced cell death has been recently described in the context of ferroptosis [63, 64]. The second “natural” approach that can induce ferroptosis is cystine deprivation, which triggers ferroptosis through GSH depletion [27, 65]. Gao et al. [27] showed that a cysteine-free medium induces cysteine and GSH depletion and results in ferroptosis of the HT1080 cell line.

Ferroptosis can also be pharmacologically induced by inhibition of GPX4 activity with the commonly used inhibitor RSL [59]. Also, the suppression of GSH synthesis by GCL inhibition with buthionine sulfoximine (BSO) in some cases represents a successful approach in ferroptosis induction [55].

**Figure 1:**
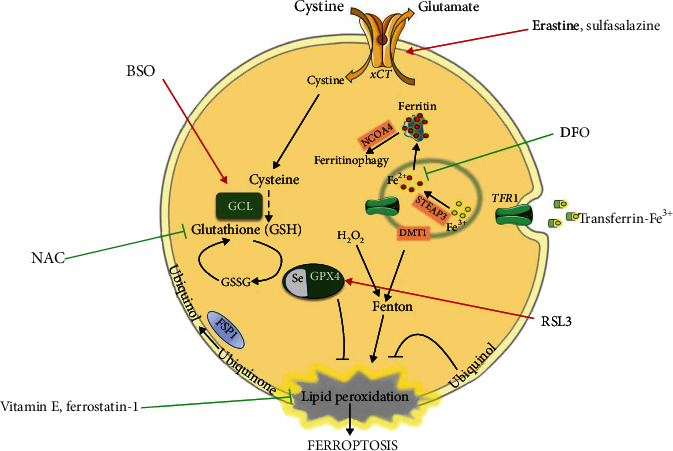
The main cellular pathways related to ferroptosis and potential targets for its manipulation (induction or inhibition). The main regulatory pathway of ferroptosis involves the cysteine-GSH-GPX4-lipid peroxide axis. Cysteine is transported into the cell via xCT in its oxidized form (cystine). One of the main roles of cysteine is the synthesis of glutathione (GSH), a process in which the key-limiting step is catalysed by glutamate-cysteine ligase (GCL). GSH serves as a cofactor of glutathione peroxidase 4 (GPX4), which reduces lipid peroxides to their alcohol form. Alternatively, lipid peroxides can be reduced by ubiquinol residing in the membrane compartments of the cell. In this way, produced ubiquinone is reduced back to its alcohol form by the action of ferroptosis-suppressor protein 1 (FSP1). The central event in the lipid peroxide production pathway is the Fenton reaction, a reaction between Fe^2+^ and H_2_O_2_. The iron is imported into the cell as an iron-loaded transferrin-transferrin receptor 1 (TFR1) complex via receptor-mediated endocytosis. In the endosome (an acidic environment), free Fe^3+^ is converted to Fe^2+^ by the transmembrane metalloreductase STEAP3 and released into the cytoplasm via the divalent metal transporter 1 (DMT1). Red arrows represent potential activators of ferroptosis: (i) inhibitors of xCT (erastin, sulfasalazine), (ii) the inhibitor of GSH biosynthesis and GCL (buthionine sulfoximine (BSO)), and (iii) the inhibitor of GPX (Ras-selective lethal 3 (RSL3)). Green arrows represent potential ferroptosis inhibitors: (i) alternative source of cysteine (N-acetylcysteine (NAC)) and (ii) lipid peroxide scavengers (vitamin E, ferrostatin-1). This figure was created using Servier Medical Art templates, which are licensed under the Creative Commons Attribution 3.0 Unported License (https://smart.servier.com).

In addition, an important regulatory point and therefore a target for ferroptosis modulators is the metabolism of iron, which is, as previously mentioned, a crucial player in the initiation of lipid peroxidation (Fenton reaction). Thus, the imbalance of intracellular iron homeostasis in favour of iron overload is pivotal in the induction of ferroptosis [33, 66, 67], while iron chelators such as deferoxamine (DFO) have been used for its inhibition [35, 68]. The molecular machinery dedicated to ferroptosis with possible targets for its modulation is presented in Figure 1.

Morphological changes related to ferroptosis also make this type of cell death unique and recognizable when compared to other types of cell death—apoptosis, necrosis, and autophagy. Ferroptosis is characterized by the so-called “ballooning” phenotype [69, 70], observed as a characteristic rounded morphology of the cell surface. This seems to be the result of several morphological alterations, including destabilization of the plasma membrane, disturbed proteostasis, and rearrangement of the cytoskeleton [71–73]. Also, ferroptotic cells exhibit extensive ultrastructural changes in the mitochondria, with the mitochondria being smaller than normal with an increased bilayer membrane density [12, 37]. There are reduction in volume, disruption of the outer mitochondrial membrane, and loss of mitochondrial cristae [44, 69]. This is clearly different when compared to apoptosis, where no significant changes in mitochondrial structure are observed, and to necrosis, in which the swelling of organelles, including the mitochondria, has been observed [23]. In addition, none of the characteristic morphological features related to necrosis (cytoplasmic swelling, plasma membrane rupture), apoptosis (chromatin condensation and margination), or autophagy (formation of double-membrane enclosed vesicles) have been associated with ferroptosis [12, 13, 19, 36].

The fact that ferroptosis cannot be inhibited by the classical inhibitors of apoptosis, autophagy, and necrosis [44] establishes the uniqueness of ferroptosis, not only from apoptosis but also from other types of cell death.

## 3. Mitochondria-Dependent Pathways in Ferroptosis

Although knowledge about the mechanisms mediating ferroptosis is constantly growing, there is still no consensus regarding the specific contribution of each organelle to the initiation and execution of this type of cell death. Current data argue that lipid peroxidation localized in the endoplasmic reticulum, lysosomes, Golgi apparatus, and mitochondria can be involved in signalling related to ferroptosis [20].

**Figure 2:**
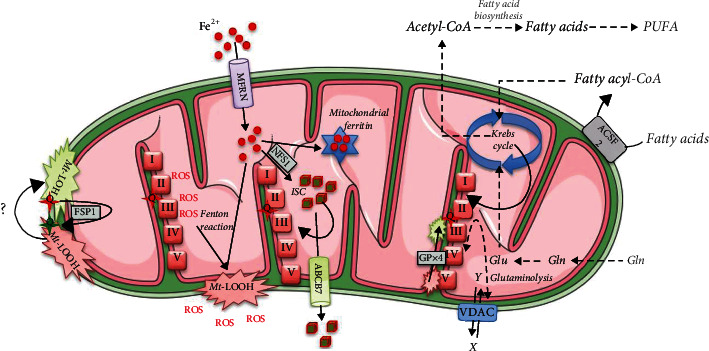
Potential mitochondrial targets for ferroptosis manipulation. Mitochondrial oxidative phosphorylation (OXPHOS) is the main cellular source of reactive oxygen species (ROS). In the presence of ferrous ions (Fe^2+^), ROS can induce the production of lipid peroxides (LOOH) in the membrane compartment of the mitochondria (Fenton reaction), thereby compromising the structure and function of the cellular powerhouse and leading to further propagation of oxidative damage within the cell. The transport of iron ions into mitochondria is achieved by mitoferrin-1/2 (MFRN), a member of the mitochondrial solute carrier family of proteins. Once inside the mitochondria, ferrous ions are used for many different purposes, such as the synthesis of heme or iron-sulphur clusters (ISCs) and key prosthetic groups of a variety of enzymes, including the TC complexes. From the standpoint of ferroptosis, the NFS1 and ABCB7 transporters, respectively, responsible for the synthesis and export of ISCs from the mitochondria to the cytosol appear to be important (see text). The excess of free iron is sequestered in mitochondrial ferritin to prevent potentially harmful effects of iron-induced oxidative damage. One possibility which remains to be examined is the potential antioxidant role of ubiquinol and ferroptosis-suppressor protein 1 (FSP1) against accumulated LOOH in the mitochondrial membrane compartment (the question mark in the scheme). The presence and protective role of glutathione peroxidase 4 (GPX4) at the side of cytochrome c release have been shown. Aside from the classical redox active species, mitochondrial metabolic intermediates appear to be involved in cell destiny, pushing it towards ferroptosis. The mitochondria-localized tricarboxylic acid (TCA) cycle is a central hub regulating fatty acid breakdown and synthesis, as well as the flux through OXPHOS. Thus, the TCA cycle could be seen as the major regulating point of ferroptosis through (i) the regulation of ROS production, (ii) the regulation of ATP production, and (iii) the regulation of the production of the precursors (acetyl-CoA units) for the synthesis of ferroptosis executors—polyunsaturated fatty acids (PUFA; #NB: the regulation of acetyl-CoA incorporation into fatty acids is regulated by cytoplasmic enzymes). The voltage-dependent anion channel (VDAC), as one of the central players in the import/export of many different ions/metabolites (X/Y), energy regulation, and ion and intermediate balance across two sides of the mitochondrial membrane, also appears to be involved in the regulation of ferroptosis (see text). ACSF2: mitochondrial medium-chain acyl-CoA ligase; Glu: glutamate; Gln: glutamine. This figure was created using Servier Medical Art templates, which are licensed under the Creative Commons Attribution 3.0 Unported License (https://smart.servier.com).

As an integrative place for many intracellular and extracellular signals, the mitochondria play a central role in determining cell destiny [3]. Their essential role in the regulation of apoptosis and necrosis has been confirmed through decades of examination of these types of programmed cell death [3, 4]. Although changes in mitochondrial morphology represent one of the criteria for the definition of ferroptosis and its distinction from other types of cell death [12], as we stated above, the contribution of mitochondrial metabolism to the triggering and execution of ferroptosis is still a matter of debate. Several aspects of mitochondria-related metabolism predispose this organelle to the induction of ferroptosis, including metabolism of energy substrates (especially lipids) and accompanying ROS production, as well as the metabolism of amino acids (glutamine) and iron (summarized in Figure 2).

Mitochondria play an important role in lipid metabolism. Citrate synthase (CS) and acyl-CoA synthetase family member 2 (ACSF2), which are localized in the mitochondria, regulate fatty acid activation and synthesis and thus provide precursors for lipid peroxidation. These enzymes have been recognized as important mediators of erastin-induced ferroptosis [12]. In addition to fatty acids, the metabolism of glutamine, a nonessential amino acid, might provide precursors for the tricarboxylic acid (TCA) cycle, especially in states of increased metabolic demand such as cancer [74]. Enzymes important for the catalysis of glutaminolysis are localized in the mitochondria. Regardless of the source of the precursors, the increase in TCA and oxidative phosphorylation (OXPHOS) leads to ROS accumulation in the mitochondria, which supports their active role in triggering ferroptosis [75]. The electron transport chain- (ETC-) mediated ROS production has been described as important for cell death in the state of GSH depletion, such as treatment with glutamate and cystine deprivation [15, 27, 76]. Tan et al. [15] have shown that ROS production and the death of HT22 cells induced by glutamate (subsequently named oxytosis) can be inhibited by ETC inhibitors or uncouplers (FCCP, carbonyl cyanide-p-(trifluoromethoxy) phenylhydrazone) that dissipate the mitochondrial membrane potential [77]. Similar changes have been shown recently in the case of ferroptosis induced by cystine depletion [27]. Important data relevant to this issue were published recently by Homma et al. [30]. The authors provided direct evidence that the superoxide anion radical produced by complex III is pivotal for cysteine starvation-induced ferroptosis; they found that the specific inhibitor of complex III, but not of complex I, protects mouse hepatoma Hepa 1-6 cells from ferroptosis in this condition.

In addition to the nonenzymatic production of ROS in OXPHOS, some enzymes localized in the mitochondria such as monoamine oxidase, NADPH oxidase 4 [78], *α*-ketoglutarate dehydrogenase, and *α*-glycerophosphate dehydrogenase [79] could contribute to increased ROS production in mitochondria. These enzymes have been proposed as mediators of RSL3-induced ferroptosis since RSL3 increases the level of the mitochondrial-specific ROS probe MitoSOX [25, 35], but this effect is not inhibited by ETC inhibitors [27].

Mitochondria also play an important role in iron metabolism. There are several iron homeostasis-regulating molecules in the mitochondria, including mitoferrin, the ABC transporter, and VDAC, all of which have been connected to ferroptosis (reviewed by Wang et al. [26] and Battaglia et al. [65]). VDAC is an important mitochondrial porin that regulates the influx and efflux of many mitochondrial metabolites in addition to iron and thereby several aspects of mitochondrial metabolism, including mitochondrial membrane potential, Ca^2+^ overload, and ROS production [80]. VDAC has been recognized as a target of some antiferroptotic approaches such as erastin, erastin-like lead compounds [36, 37], and RSL5 [81]. Cells expressing more VDAC protein were more sensitive to erastin [36]. Calcium chelators effectively counteract erastin-induced cell death in LUHMES cells [82], confirming hyperpolarization of the mitochondrial membrane in ferroptosis. It was also shown that the suppression of two genes encoding the critical steps of mitochondrial iron-sulphur cluster (ISC) biosynthesis, NFS1 and ABCB7, leads to an increase in intracellular free iron and promotes cell death by ferroptosis [83].

Apart from the pathways connected to the fundamental role of mitochondria in cells, there are some specific factors that might link mitochondria and ferroptosis. One of these is BID, a proapoptotic protein of the Bcl-2 family, whose translocation into the mitochondria has been shown to mediate erastin-induced ferroptosis in neuronal cells [25]. Also, the flavoprotein apoptosis-inducing factor mitochondria-associated 2 (AIFM2) has recently been recognized as an important antiferroptotic factor involved in maintaining the antioxidant activity of coenzyme Q in the membrane compartment of the cell and has been renamed ferroptosis suppressor protein 1 (FSP1) [14, 84]. It is interesting to note that FSP1 could be seen as an alternative to the GSH-GPX4 part of the ferroptosis-regulating axis, providing protection against the accumulation of lipid peroxides even in conditions of GSH depletion [70].

Growing evidence points to the relevance of oxidation of the mitochondrial membrane in ferroptosis induction/execution [19, 28]. Friedmann Angeli et al. [19] provided direct evidence that the oxidation of mitochondrial membranes is important for the execution of ferroptosis. They found an accumulation of oxidized mitochondria-specific phospholipid cardiolipin in the kidney of GPX4-knockout mice. In this context, mitochondrially localized GPX4 plays an important role in protection from ferroptosis [85]. The presence of GPX4 in mitochondria at the side of cytochrome c release and its protective role in apoptosis have been previously shown [86].

## 4. The Role of Mitochondria in Ferroptosis Induction in Cancer

The possibility to interfere with ferroptosis signalling pathways and to manipulate this type of cell death might be of great importance in cancer therapy. There is hope among cancer researchers that ferroptosis induction could be a successful anticancer approach since many cancer types that are resistant to conventional chemotherapy appear to be sensitive to ferroptosis [87, 88]. An important example is pancreatic ductal adenocarcinoma (PDAC) that is resistant to many cancer therapies. As was recently reported in *in vitro* and tumour xenograft studies [68, 89], the inhibition of xCT either with erastin or by genetic ablation sensitizes PDAC cells to ferroptosis and could thus present a promising therapeutic strategy for this type of cancer. It is likely that the sensitivity of cancers to common genetic or pharmacological approaches for manipulation of ferroptosis differs between various types of cancer, with the highest sensitivity observed in brain tumours [90, 91]. However, the precise mechanisms that determine the sensitivity of cancer cells to ferroptosis induction are not fully elucidated. On the other hand, it is well known that mitochondria underlie the phenotypic and metabolic plasticity of cancer cells and are responsible for the resistance of cancer cells to regulated types of cell death, especially apoptosis [92]. Considering the multifaceted role of mitochondria in cancers (reviewed in Badrinath and Yoo [93]), more mitochondria-based cancer therapies are needed to guarantee fully beneficial outcomes in cancer treatment. In that context, the involvement of mitochondria and mitochondrial metabolism in ferroptosis deserves more attention.

In the initial characterization of ferroptosis, Dixon et al. [12] reported that mitochondrial DNA- (mtDNA-) depleted *ρ*^0^ (Rho0) cells remained sensitive to oxidative stress and ferroptosis induction in the fibrosarcoma cell line, HT1080. This was confirmed in their work on mitochondria-depleted HT1080 cells generated by a mitophagy protocol [29, 94]. These cells exhibited similar sensitivity to ferroptosis-modulating agents (the inducers erastin and RSL1 and inhibitors ferrostatin-1 and iron chelators) as their counterparts harbouring mitochondria. Furthermore, Ye et al. [95] showed that erastin-induced ferroptosis in a leukaemia cell line did not involve changes in mitochondria-mediated ROS generation. Although the data suggest that mitochondria are not required for cancer-associated ferroptosis, these results are somewhat surprising considering all the above-discussed aspects of mitochondrial metabolism that should interfere with the ferroptotic process.

It is likely that contradictory results on the involvement of mitochondria in ferroptosis are due to different methods for measuring both mitochondrial function and cell death, as well as differences between cancer cells. Using Rho0 cells might not be the best method for investigating the significance of mitochondria in cell death processes because Rho0 cells are generated by long-term cultivation with ethidium bromide that frequently induces damage of nuclear DNA [96]. This could affect the response of the cells to oxidative stress-inducing agents, including different ferroptosis inducers. Furthermore, it seems likely that there are differences between various Rho0 cancer cell lines in their sensitivity to ferroptosis. In contrast to the Rho0 fibrosarcoma cell line that shows similar sensitivity to ferroptosis-inducing agents to that of parental cells (as described above and in Dixon et al. [12]), Rho0 cells established from human cervical cancer and oral squamous cell carcinoma cell lines display more sensitivity to H_2_O_2_-induced ferroptosis, along with increased levels of ^∙^OH, Fe^2+^, and lipid peroxidation [97, 98]. These results have highlighted the relevance of mitochondrial dysfunction in ferroptosis induction in cancer and must be considered during the development of new anticancer ferroptosis-based approaches.

Increasing evidence suggests that mitochondrial metabolism may be of functional relevance to ferroptosis progression and final execution in cancer cells. For example, acyl-CoA synthetase long-chain family member 4- (ACSL4-) dictated ferroptosis in breast cancer cells is accompanied by changes in mitochondrial morphology [14]. Literature also suggests that erastin-induced anticancer activity might be mediated by mitochondria [36, 37]. For example, DeHart et al. [36] have shown that erastin-induced cell death in a hepatocarcinoma cell line involves VDAC opening and mitochondrial hyperpolarization, followed by increased ROS production, mitochondrial dysfunction, and finally a collapse of the transmembrane potential. Using RNAi-based loss-of-function screening in lung cancer cell cultures and tumour xenografts, Alvarez et al. [83] showed that the suppression of two genes encoding critical steps of mitochondrial ISC biosynthesis, NFS1 and bcl7, leads to iron release from intracellular stores and promotes cell death by ferroptosis. ISCs, as critical components of the OXPHOS complexes and many other metabolic enzymes, are fundamental to the normal functioning of mitochondria, especially at high concentrations of O_2_. The downregulation of NFS1 (i) induces loss of essential cell functions due to cofactor depletion and (ii) sensitizes cells to ferroptosis by activating the iron starvation response and iron overload.

It was recently shown that the mitochondria only play a role in cysteine deprivation-induced ferroptosis but not in ferroptosis induced by GPX4 inhibition [27]. As the authors explained, this is probably because the mitochondrial function upstream of GPX4 promotes the exhaustion of GSH under cysteine deprivation conditions. In contrast to Dixon's laboratory, Gao et al. [27] observed that depletion of the mitochondria through parkin-mediated mitophagy or inhibition of OXPHOS dramatically decreased the sensitivity of cells to cysteine deprivation-induced ferroptosis in a human fibrosarcoma cell line. Their work also highlighted the importance of glutaminolysis as an indispensable part of cysteine deprivation-induced ferroptosis. In most cancer cells, the rate of glutaminolysis is increased to meet their bioenergetic requirements [73]. However, this could make cancer cells more vulnerable to ferroptosis induction. Under cysteine deprivation, glutaminolysis promotes mitochondrial respiration and rapid exhaustion of GSH by GPX4, rendering these cells dependent on the cysteine-GSH supply and thus prone to ferroptosis. Recently published data also speak in favour of the significance of mitochondrial metabolism in the execution of ferroptosis in cancer cells. Namely, it has been suggested that the rerouting of tumour cell metabolism from glycolysis to OXPHOS could make cells more vulnerable to GSH depletion and ferroptosis [99].

A significant amount of data suggests that mitochondria and mitochondria-related signalling could be a promising target in terms of ferroptosis induction as an anticancer therapeutic strategy. However, further studies are needed to establish specific targets and approaches for their manipulation, as well as the specificity and sensitivity of different tumours to such manipulations.

## 5. Mitochondria and Ferroptosis in Cardiovascular Diseases

Myocardial infarction and heart failure are cardiovascular diseases with the highest mortality rates worldwide [10]. A crucial pathogenic factor in the development of both pathologies is the loss of terminally differentiated cardiomyocytes [10, 100, 101]. Pharmacological and genetic manipulations indicate that cardiomyocyte cell death is the central event in the pathogenesis of both diseases [10, 100, 101]. In addition to apoptosis and necrosis, ferroptosis has recently emerged as an important cause of cardiomyocyte death, at least in I/R heart injury [31, 33, 102]. Moreover, the mechanisms of cardiomyocyte ferroptotic cell death appear to involve mitochondria-dependent pathways [31, 33]. This is quite reasonable considering that mitochondria have previously been described as essential to proper cardiomyocyte functioning [103, 104].

A study performed by Fang et al. [33] demonstrated that the increase in free iron as the result of heme degradation drives ferroptosis of cardiomyocytes in apoptosis- and/or necroptosis-defective *Ripk3^−/−^*, *Mlkl^−/−^*, or *Fadd^−/−^Mlkl^−/−^* mice exposed to the DNA-damaging agent doxorubicin or in the I/R condition. It was shown that excess free iron accumulates in the mitochondria, not in the cytoplasm, and causes lipid peroxidation specifically in mitochondrial membranes. The authors further showed that the inhibition of pathways that mediate mitochondria-driven ferroptosis (after treatment with a specific mitochondria-targeted antioxidant, MitoTEMPO) significantly ameliorated doxorubicin-induced lipid peroxidation and cardiac ferroptosis. Moreover, MitoTEMPO but not TEMPO, a nonspecific antioxidant, significantly reduced doxorubicin-induced cardiomyopathy, assessed by measuring the heart/body weight ratio, serum levels of myocardial enzymes, and cardiac hypertrophy biomarkers. These data clearly showed that oxidative damage of mitochondria is the major consequence of iron overload in I/R-induced heart damage and suggest that the decrease in mitochondrial iron accumulation and/or inhibition of lipid peroxidation may be cardioprotective during both acute and chronic cardiac I/R.

Apart from the above-described role of mitochondria in cardiac I/R injury-associated cardiomyocyte ferroptosis, another study performed by Feng et al. [31] revealed additional mitochondria-dependent mechanisms that could be involved in the execution of ferroptosis. The authors showed that postischemic application of liproxstatin-1 to the mouse myocardium decreased I/R injury, as shown by the decreased size of the infarct-affected area. Importantly, they found that the observed cardioprotective effects of liproxstatin-1 were achieved through maintenance of mitochondrial structural and functional integrity. The precise mechanism of the beneficial effects of liproxstatin-1 on mitochondria involves the reduction of VDAC1 level and its oligomerization, without affecting VDAC2/3, as well as a decrease in mitochondrial ROS production by the ETC complex I (NADH:ubiquinone oxidoreductase). These results are in agreement with studies postulating that mitochondrial ROS generation is a significant contributor to ferroptosis [105, 106]. This contrasts with the often-represented standpoint that mitochondrial damage is a secondary event in ferroptosis [13, 80] and also opposes studies that did not observe changes in complex I ROS production in erastin-induced or ferrostatin-inhibited ferroptosis [12, 63], suggesting that the impact of mitochondrial ROS on ferroptosis might be dependent on the specific inducer or inhibitor of ferroptosis used. In any case, the results of Feng et al. [31] revealed the antiferroptotic and cardioprotective effects of liproxstatin-1 via direct mitochondrial pathways.

These discoveries raise the possibility of attenuating cardiomyocyte cell death through the manipulation of the mitochondrial signalling pathways and may provide novel therapies for common and highly lethal heart diseases.

## 6. Mitochondria and Ferroptosis in Neurodegenerative Diseases

All neurodegenerative diseases are characterized by the progressive and irreversible loss of neurons from specific regions of the central and peripheral nervous systems, followed by a decline in neuronal, motor, and/or cognitive functions [107]. It was shown that cellular death driven by increased levels of iron, ROS, and consequent lipid peroxidation—ferroptosis—is an important contributor to neural cell death in Alzheimer's, Parkinson's, and Huntington's diseases, multiple sclerosis, and acute brain injury caused by cerebral ischemia, haemorrhagic insults, or brain trauma [108–111]. In addition, more recent studies have suggested that mitochondrial damage might be the ultimate step in ferroptosis-related oxidative cell death in neurodegeneration [112]. It was shown that brain regions with the highest levels of iron and ROS accumulation are associated with excessive mitochondrial damage, clearly linking the loss of mitochondrial integrity and function to oxidative neuronal death and neurodegeneration [112].

The first suggestion that the process of ferroptosis in neurodegenerative diseases is regulated by mitochondria came from the research of Gao and Chang [21] who showed the involvement of mitochondrial ferritin in the regulation of brain iron homeostasis and oxidative neuronal cell death, while Wang et al. [32] demonstrated the protective role of mitochondrial ferritin in erastin-induced ferroptosis. Iron accumulation in mitochondria is also found in ferroptosis related to Friedreich's ataxia where the mitochondria-targeted antioxidant XJB-5-131 was found to be effective in inhibiting ferroptosis [34].

The involvement of mitochondria in neurodegeneration-associated ferroptotic cell death is further demonstrated in PC12 cells where the induction of ferroptosis by tert-butyl hydroperoxide (t-BHP) was accompanied by a decrease in the mitochondrial membrane potential, decreased ATP production, and an increase in mitochondrial ROS [112]. Moreover, in the same model system, it was observed that apart from suppressing ferroptosis, ferrostatin-1 also “repaired” mitochondrial dysfunction, strongly indicating that mitochondrial dysfunction is closely related to ferroptosis [112]. These results are in line with the data of Jelinek et al. [35] who reported that in addition to ferrostatin-1, other ferroptosis inhibitors are also capable of preventing mitochondrial damage in neuronal HT-22 cells and of decreasing ferroptosis induced by RSL3-dependent inhibition of GPX4 and the generation of damaging free radicals. The authors [35] established that in neuronal HT22 cells, RSL3 mediates the concentration-dependent inhibition of GPX4 followed by lipid peroxidation, enhanced mitochondrial fragmentation, reduction of mitochondrial membrane potential, and respiration, while ferroptosis inhibitors, such as DFO, ferrostatin-1, and liproxstatin-1, but also CRISPR/Cas9 Bid knockout and the BID inhibitor BI-6c9, protected against RSL3 toxicity. They presented new data that the mitochondria-targeted ROS scavenger mitoquinone (MitoQ) preserved mitochondrial integrity and function, as well as cell viability despite significant loss of GPX4 expression accompanied by an increase in general lipid peroxidation after exposure to RSL3.

Apart from the *in vitro* data indicating that rescuing mitochondrial integrity and function might be effective in the prevention of ferroptosis in neural cells, the involvement of mitochondria and ferroptosis in neurodegenerative diseases has been supported by results obtained in studies performed *in vivo* on experimental animals [113–115]. It was shown that the expression of all three GPX4 isoforms is reduced in both the brain of multiple sclerosis patients and the spinal cord of animals with an experimental autoimmune encephalomyelitis (EAE) [115]. The detected decrease in GPX4 in EAE was accompanied by morphological changes in the mitochondria characteristic for ferroptosis, including an irregular matrix, disrupted outer membrane, and reduced or absent cristae [115]. In addition, in the experimental model of arsenite-induced neuronal cell death that was shown to be associated with various neurodegenerative diseases such as Alzheimer's and Parkinson's, as well as amyotrophic lateral sclerosis (ALS), it was shown that the loss of neurons is caused by the accumulation of ROS and characteristic ferroptotic events, including mitochondrial VDAC-related pathways [114]. Although specific identification of ferroptosis *in vivo* is hampered by lack of specific biomarkers, it was shown that conditional ablation of GPX4 to the forebrain neurons of adult mice causes hippocampal degeneration resembling Alzheimer's disease and cognitive impairments accompanied by lipid peroxidation and mitochondrial impairments consistent with ferroptosis [113]. This can be partly rescued by inhibitors of ferroptosis, confirming ferroptosis involvement.

All the above data indicate that rescuing mitochondrial integrity and function might be effective in the prevention of ferroptosis in neural cells and provide a new approach in targeting cell death in neurodegenerative conditions. Therefore, mitochondria-dependent mechanisms of ferroptosis in the nervous system need to be fully explored in future research.

## 7. Ferroptosis in Other Pathologies and the Involvement of Mitochondria

The list of pathologies that involve ferroptosis as an underlying mechanism of their aetiology and progression is growing, as is knowledge about the signalling pathways regulating this type of cell death. In this context, ferroptosis has been established as a contributing factor to tissue damage in ischemic injury of the kidney and in liver diseases [18, 19, 63], in addition to the previously described contribution of apoptosis and necrosis [116–119]. Ferroptosis is also involved in liver haemochromatosis [120] and mediates the activity of anticancer drugs commonly used in hepatocellular cancer therapy (for example, sorafenib) [121]. Although it was initially suggested that ferroptosis was responsible for acetaminophen- (APAP; paracetamol) induced liver injury [122, 123], recently published papers argue against this hypothesis [124, 125]. Jaescke et al. [125] critically reviewed this issue and suggested that there is no evidence for a quantitatively sufficient amount of lipid peroxides to cause cell death, and thus, APAP hepatotoxicity is not caused by ferroptosis. At the same time, the authors did not exclude the significance of mitochondrial oxidative stress (peroxynitrate) in APAP-induced hepatotoxicity.

Mitochondrial dysfunction plays a critical role in the pathogenesis of different liver and kidney diseases [126, 127], but it is usually linked with tissue injury due to apoptosis, rarely ferroptosis. One of these rare examples comes from studies of an *in vitro* model of kidney I/R injury that showed that erastin-induced ferroptosis in human kidney-2 (HK-2) cell lines was mediated by changes in mitochondrial metabolism, such as hyperpolarization of the mitochondrial membrane [128] or interaction with a specific mitochondrially localized protein ALR (augmenter of lipid regeneration) [129]. Acute renal failure induced by GPX4 inactivation is accompanied by the accumulation of oxidized cardiolipin in mitochondria and consequent ferroptosis [19].

Scarce data on the role of mitochondria in liver and kidney disease-related ferroptosis, along with the significant impact of this process on their pathogenesis, has opened the field for further examination, with the final aim of producing novel mitochondria-targeted antiferroptotic approaches for the treatment of different pathologies.

## 8. Ferroptosis in Metabolic Disorders: Possible Implication in Diabetes

In addition to the previously mentioned diseases, mitochondrial dysfunction plays an important role in the pathogenesis of metabolic disorders, such as obesity, metabolic syndrome, and diabetes. The involvement of cell death (apoptosis, necrosis, and autophagy) in diabetes and its link with mitochondrial dysfunction are well documented [117, 130, 131]. To date, there are no studies confirming the role of ferroptosis in the regulation of *β*-cell mass and the pathology of tissues in the diabetic state. However, there are several peculiarities that characterize the diabetic phenotype, and the pancreas itself, that speak in favour of the possible involvement of ferroptosis in the aetiology and pathogenesis of this disease.

First, the production of ROS is increased in the diabetic state, especially at the mitochondrial level, due to (i) hyperglycaemia, (ii) hyperlipidaemia, (iii) hyperinsulinemia, and (iv) iron overload [132–135]. Along with the increase in the production of ROS, the antioxidative defence is decreased in many diabetes-targeted tissues [136, 137]. This leads to an increase in oxidative pressure commonly seen as an increase in the level of lipid peroxidation end products, suggesting that the GPX4-GSH antioxidant axis is compromised in this condition [138, 139]. Along with this, *β*-cells are extremely sensitive to oxidative insults due to the intrinsically low expression and activity of antioxidative enzymes, including GPX [140]. In line with the hypothesis that *β*-cells are prone to ferroptosis, the recent paper from Bruni et al. [68] argues that the viability and function of pancreatic *β*-islets *in vitro* are highly compromised in the presence of ferroptosis-inducing agents. The authors showed that human islets isolated for transplantation are susceptible to erastin- and RSL3-induced ferroptosis and that this can be suppressed by ferrostatin-1.

With such a background as the starting point, the examination of ferroptosis in diabetic conditions represents an intriguing and important scientific field that holds great potential for the improvement of antidiabetic therapy. Our efforts along these lines are in progress.

## 9. Mitochondria-Specific Strategies and Future Directions in Targeting Ferroptosis

According to the presented information related to mitochondria and ferroptosis reviewed herein, the targeting of mitochondrial metabolism presents a new concept for effective (i) antiferroptotic (cardiovascular and neurodegenerative diseases) and (ii) proferroptotic (cancer) strategies.

Considering the studies summarized in the present review, an antiferroptotic strategy that deserves more attention could be the use of specific mitochondria-targeted antioxidants. The requirement for organelle-targeted antioxidants and even more targeting of the specific site of ROS production [76] has emerged after the failure of most antioxidant therapies to achieve significant outcomes in clinical trials [141, 142]. A possible reason for this is that after *in vivo* application, only a small amount of antioxidant reaches cellular organelles, including the mitochondria. Thus, great effort has been invested in the design and testing of mitochondria-targeted antioxidants in a wide range of pathological conditions where mitochondrial dysfunction followed by increased mitochondrial ROS production has a significant impact [143–147].

To date, several mitochondria-targeted antioxidants have shown promise in the treatment of human pathologies such as neurodegenerative diseases [148], ischemia/reperfusion [149, 150], diabetes [151–153], hypertension [154, 155], and renal diseases [156, 157]. This list includes MitoVitE, MitoQ, MitoTEMPO(L), and MitoLipoic acid. The beneficial effects of such compounds are commonly seen as a decrease in oxidative stress in the mitochondria and consequent improvement in the pathologies, reflected through specific parameters including tissue damage and cell death. In this context, apoptosis, as a form of mitochondria-related cell death, is well studied, and the expected benefit of targeted antioxidants has been confirmed in many cases [154, 158, 159].

Judging by the growing evidence for the involvement of mitochondrial ROS accumulation in ferroptosis in different pathological states as reviewed here, we believe that the use of antioxidants that specifically target these organelles has promising potential as an antiferroptotic strategy in many pathologies. To date, there are only a few studies that deal with the effects of mitochondria-targeted antioxidants on ferroptosis and ferroptosis-related events. In most of these studies, these antioxidants have been used as tools for examining the contribution of mitochondria to the process of ferroptosis *per se* [30, 35, 36], while in some recently published papers, their protective effects in several pathological conditions have been ascribed to the suppression of ferroptosis [33, 160]. Importantly, these data have paved the way for future research directed at the development of antiferroptotic strategies in many pathological conditions.

In addition to mitochondria-targeted antioxidants, mitochondria-permeable iron chelators could be a promising approach for the suppression of mitochondrial iron overload and the consequent process of ferroptosis. DFO has commonly been used for testing the sensitivity of ferroptosis-related events in the modulation of intracellular iron. DFO is not very membrane-permeable and cannot effectively chelate intracellular iron in some tissues such as the heart [161, 162] and brain [163]. Although Dixon et al. [12] showed in the first study defining ferroptosis that membrane-permeable iron chelators such as 2,2-bipyridyl (2,2-BP) provide adequate protection against ferroptotic cell death, such that modulators of iron concentrations have not been sufficiently exploited in subsequent studies of this type of cell death. We believe that these avenues for targeting ferroptosis require attention as a therapeutic strategy in many pathologies where iron overload in the mitochondria plays a significant role, at least in neurodegenerative diseases and cardiomyopathies [164, 165].

In the context of cancer therapy, an opposite approach should be applied, one that is directed to the induction of ferroptosis by targeting mitochondria. A constant aim in cancer therapy is to overcome resistance to cell death in cancer cells due to their specific mitochondrial phenotypes [166]. Multiple strategies have been developed thus far, including those that target the ETC and OXPHOS functions, glycolysis, the TCA cycle, ROS homeostasis, and permeability transition pores [167, 168]. The antitumour activity of many of them interferes with the pathways that lead to increased ROS production, and they can induce apoptosis with varying success. Given the versatile roles of mitochondria in cancer cells, more mitochondria-based therapies are warranted. In this context, proferroptotic mitochondria-related cancer therapy promises to overcome the drawbacks of traditional therapies mediated by apoptosis and should be extensively studied in the future. Among the proferroptotic agents, erastin has been the most extensively studied as an anticancer agent. It acts by opening the VDAC and the consequent increase in mitochondrial ROS production followed by ferroptotic-related events [169].

Thus, we suggest that the commonly used mitochondria-directed approaches should now be exploited in a completely new context which is focused on the process of ferroptosis. This could be a promising strategy in overcoming major problems in the pathologies mentioned in the present review and dysregulation of cell death signalling pathways and in improving existing therapies.

## 10. Conclusion

With the highest mortality rates worldwide, cancer, neurodegenerative, cardiovascular, and hematologic diseases, and ischemia/reperfusion injury of the heart, brain, and kidney occupy the attention of scientists searching for effective novel approaches for treating these high-impact pathologies. Extensive research that has been performed during the last years indicates that ferroptosis might be involved in the aetiology of all the above-mentioned diseases and that mitochondrial pathways are likely involved in the regulation of ferroptosis. Therefore, mitochondria and mitochondria-related signalling are promising targets for modulating ferroptosis and warrant further examination with the aim of obtaining improved understanding and treatment of these pathologies. We have specifically highlighted here the issues that should be addressed in future research and that may eventually be considered potential targets in pro-/antiferroptotic therapy of the mentioned diseases, as well as in other pathological conditions accompanied by increased cell death and/or impaired mitochondrial function.

## Data Availability

No data were used to support this study.
